# Performance metrics and reference standard uncertainty in the CT-LVEF study

**DOI:** 10.1093/ehjdh/ztag106

**Published:** 2026-06-30

**Authors:** Emaan Fatima

**Affiliations:** Dow University of Health Sciences, Karachi 74200, Pakistan

We read with great interest the recently published article by Raikhelkar *et al*.^1^ describing an AI model capable of detecting abnormal left ventricular ejection fraction (LVEF) using non-contrast chest CT. The opportunistic screening paradigm is compelling, and the multi-institutional validation is a definite strength. However, we wish to highlight two points that we believe materially affect the interpretation of reported performance.

Firstly, the manuscript reports an overall F1-score of 0.822 (CU hold-out) and 0.812 (WCM external validation) and uses a ‘weighted F1’ of 0.808/0.797 to argue the model outperforms two thoracic radiologists (Table 7). However, the confusion matrices in Fig. 4d,e allow the class-specific performance to be recovered: recall for abnormal EF (positive, clinically actionable class) is 259/846 = 0.31 and precision is 259/433 = 0.60, resulting in a positive-class F1 of 0.41 in the CU hold-out set, and recall is 263/1415 = 0.19 and precision is 263/365 = 0.72, resulting in a positive-class F1 of 0.30 in WCM external validation (*Table 1*). These numbers can be fully derived from the authors’ own data and differ by about a factor of two from the headline values reported. This divergence is due to prevalence weighting: since abnormal EF accounts for only ∼16–17% of the sample, the weighted F1 metric is driven predominantly by correct classification of the large normal-EF majority. Aggregate performance metrics in imbalanced datasets may mask clinically relevant performance in the minority positive-class.^2^ For a screening tool whose entire clinical rationale is detecting undiagnosed reduced ejection fraction, this metric is poorly suited to support the paper's central comparative claim against radiologists. In addition to the existing AUROC and decision-curve analyses, we would ask that the authors include positive-class recall, precision, and F1 (not weighted F1) as the main classification metrics in any future communication.

**Table 1:** Class-specific vs. headline performance metrics (derived from Figure 4d–e)

Cohort	Headline F1 (Reported)	Positive-class Precision	Positive-class Recall	Positive-class F1
CU hold-out	0.822	0.60	0.31	0.41
WCM external validation	0.812	0.72	0.19	0.30

A second concern relates to the quality of the reference standard itself. Table 8 shows that a notable proportion of LVEF values serving as the reference standard were not derived from discrete echocardiographic measurements but were instead recorded as ranges—indicated by terms like ‘mean’, ‘∼’, ‘<’, and ‘>’. This affects ∼26–29% of the WCM external validation cohort. The authors’ convention is followed to derive the binarized label by subtracting or adding 2.5 from a value of ‘<’ or ‘>’, and ‘mean’ ranges (e.g. ‘40–60%’) are collapsed to a single point estimate. This differs from the inter/intra-observer variability (8–21%) previously identified as a limitation: range-based recording involves structural label uncertainty that is disproportionately skewed to cases that likely have a true EF around the 50% cut-off. If the patient's true EF is 48%, the model should ideally learn to classify it as abnormal; however, if the same case is reported as ‘40–60%’ and binarized as normal per the authors’ convention, then the training signal is inverted. This concern is different from measurement variability, because it is about uncertainty in the reference standard itself. Previous methodological work has demonstrated that imposing dichotomous disease classification from uncertain or imperfect reference standards may bias estimates of diagnostic accuracy.^3^ Consequently, the influence of such uncertainty on the reported AUROC and the chosen sensitivity/specificity operating-point (64%/80%) cannot be predicted in either direction or magnitude from the authors’ general acknowledgment of measurement variability. We recommend that the authors conduct a sensitivity analysis restricted to ‘exact’ (operator ‘=’) LVEF values, or at least stratify performance by exact vs. range-derived ground truth, to assess whether the model's discriminative performance remains reliable despite this label noise.

In summary, both concerns are readily identifiable from data already published in the manuscript and do not require additional data collection or an entirely new study. Addressing the choice of performance metric and the potential impact of label granularity would substantially strengthen confidence in the comparative claims made by the authors and would enhance the value of what is otherwise an important contribution to the field.
